# Leukoerythroblastosis Mimicking Leukemia: A case report

**Published:** 2014-06

**Authors:** Aylin Canbolat Ayhan, Cetin Timur, Yusuf Ayhan, Gulsen Kes

**Affiliations:** Department of Hematology-Oncology, Education and Research Hospital, Pediatrics, Istanbul Medeniyet University Goztepe, Turkey

**Keywords:** Leukoerythroblastosis; Leukemoid reaction; Leukemia; Abscess

**To the Editor,**

Leukoerythroblastosis due to infections can resemble leukemia, differential diagnosis can be difficult. Bone marrow examination is essential for differential diagnosis. Herein we describe a patient with leukoerythroblastosis and hepatosplenomegaly associated with inguinal abscess which was difficult to distinguish from juvenile myelomonocytic leukemia. 

 A 3-month-old boy was admitted to hospital with complaints of fever, vomiting and abdominal distension. He was febrile (38 ^o^C). Liver was palpable 3 cm, spleen 4 cm below the costal margins. Enlargement of bilateral inguinal lymph nodes and a mass lesion with fluctuation in the left inguinal region were observed. Laboratory findings: Hemoglobin 3.3 gr/dl, WBC 75000/mm^3^, platelets 253000/mm^3^, serum lactate dehydrogenase (LDH): 108 1U/L, uric acid: 6.4 mg/dl, other biochemical tests were normal. C-reactive protein (CRP) 6.61mg/L. Direct and indirect Coombs tests were negative. Peripheral smear examination revealed segmented neutrophils 53%, band neutrophils 7% lympho-cytes 18%, monocytes 13%, promyelocytes 1% myelocytes 2%, metamyelocytes 4%, eosinophils 2%. Left shift was accompanied by normoblasts but erythrocyte morphology was not compatible with hemolytic anemia and there were no blasts. During his follow-up his thrombocyte count decreased to 45000/mm^3^. Abdominal ultrasound revealed hepatosplenomegaly, bilateral inguinal lymphadenomegaly (left: 34×18 mm, right: 31×11 mm) and a mass lesion (35×20 mm) in the left inguinal region. Pathological evaluation of needle aspiration of the lesion demonstrated abscess formation but we could not identify any microorganism from drainage specimen. Systemic antibiotic therapy was started. Because of the extremely elevated leucocyte count with monocytosis, circulating immature myeloid cells and nucleated red blood cells (RBCs) chronic juvenile myelomonocytic leukemia (JMML) was suspected and bone marrow aspiration performed. It did not demonstrate any blasts or monoclonality. Flow cytometric immunophenotyping excluded leukemia. Philadelphia chromosome and BCR/ABL fusion were negative. Leukocyte alkaline phosphatase (LAP) score was 54. Karyotyping was normal. All these results excluded leukemia. Bone radiographs were normal so osteopetrosis was not considered. He did not have immunodeficiency. His serum immunoglobulin G, A, M levels were normal. Analysis of lymphocyte subsets was normal. Cytomegalovirus (CMV) immunoglobulin M (IgM), EBV IgM, Parvovirus IgM, *Toxoplasma gondii* IgM, Rubella IgM, Rubeola IgM and Varicella IgM were negative; CMV and Parvovirus DNA PCR were also negative. Based on these findings our diagnosis was leukoerythroblastosis due to inguinal abscess. On the 10^th^ day of antibiotics, White blood cells (WBC) decreased to 30000/mm^3^, on the 27^th ^day to 20000/mm^3^, Platelet 154000/mm^3^. At discharge liver, spleen and lymphadenopathies were not palpable anymore. WBC was 13000 /mm^3^. Peripheral WBC count higher than 50000/mm^3^ with significant increase in early myeloid precursors is called leukemoid reaction. In leukoerythro-blastosis left shift is accompanied by RBCs^[^^1^^,^^2^^]^. Differential diagnosis of leukemoid reactions should be made with leukemias and other causes such as infections, hemorrhage, drugs, hypersensivity syndrome, myeloid growth factors, malignancy and splenectomy^[^^1^^-^^3^^]^. In 35% of patients with WBC >50000/mm^3^, leucocytosis was caused by leukemoid reaction^[^^2^^]^. In leukemoid reaction WBC count returns to normal when the predisposing factor is treated. Leukoerythroblastic reaction and presence of monocytosis is usually seen in JMML, chronic myeloid or acute monoblastic leukemias^[^^1^^]^. CMV infection could mimic JMML^[^^4^^]^. Some infections can lead to serious depression of complete blood count and cause hepatosplenomegaly. In leukemoid reaction anemia and thrombocytopenia are usually not expected. Hepatosplenomegaly, lymphadenopathy, contribution of anemia and thrombocytopenia are frequently the features of leukemia^[^^5^^]^.

 leukoerythroblastosis can be associated with infections but bone marrow examination is essential for differential diagnosis with leukemia. 
